# Characterizing upper extremity fine motor function in the presence of white matter hyperintensities: A 7 T MRI cross-sectional study in older adults

**DOI:** 10.1016/j.nicl.2024.103569

**Published:** 2024-01-24

**Authors:** Riccardo Iandolo, Esin Avci, Giulia Bommarito, Ioanna Sandvig, Gitta Rohweder, Axel Sandvig

**Affiliations:** a Department of Neuromedicine and Movement Science, Faculty of Medicine and Health Sciences, Norwegian University of Science and Technology (NTNU), Trondheim, Norway; b Stroke Unit, Department of Medicine, St Olav’s University Hospital, Trondheim, Norway; c Department of Clinical Neurosciences, Lausanne University Hospital and University of Lausanne, Lausanne, Switzerland; d Department of Neurology and Clinical Neurophysiology, St. Olav’s University Hospital, Trondheim, Norway; e Department of Clinical Neurosciences, Division of Neuro, Head and Neck, Umeå University Hospital, Umeå, Sweden; f Department of Community Medicine and Rehabilitation, Umeå University Hospital, Umeå, Sweden

**Keywords:** Aging, Neurodegeneration, Motor impairment, High-resolution MRI, Leukoaraiosis

## Abstract

**Background:**

White matter hyperintensities (WMH) are a prevalent radiographic finding in the aging brain studies. Research on WMH association with motor impairment is mostly focused on the lower-extremity function and further investigation on the upper-extremity is needed. How different degrees of WMH burden impact the network of activation recruited during upper limb motor performance could provide further insight on the complex mechanisms of WMH pathophysiology and its interaction with aging and neurological disease processes.

**Methods:**

40 healthy elderly subjects without a neurological/psychiatric diagnosis were included in the study (16F, mean age 69.3 years). All subjects underwent ultra-high field 7 T MRI including structural and finger tapping task-fMRI. First, we quantified the WMH lesion load and its spatial distribution. Secondly, we performed a data-driven stratification of the subjects according to their periventricular and deep WMH burdens. Thirdly, we investigated the distribution of neural recruitment and the corresponding activity assessed through BOLD signal changes among different brain regions for groups of subjects. We clustered the degree of WMH based on location, numbers, and volume into three categories; ranging from mild, moderate, and severe. Finally, we explored how the spatial distribution of WMH, and activity elicited during task-fMRI relate to motor function, measured with the 9-Hole Peg Test.

**Results:**

Within our population, we found three subgroups of subjects, partitioned according to their periventricular and deep WMH lesion load. We found decreased activity in several frontal and cingulate cortex areas in subjects with a severe WMH burden. No statistically significant associations were found when performing the brain-behavior statistical analysis for structural or functional data.

**Conclusion:**

WMH burden has an effect on brain activity during fine motor control and the activity changes are associated with varying degrees of the total burden and distributions of WMH lesions. Collectively, our results shed new light on the potential impact of WMH on motor function in the context of aging and neurodegeneration.

## Highlights

• WMH are prevalent in elderly and a risk factor for multiple neurological diseases.
• WMH spatial distribution differentially alters upper extremity neural activations.
• WMH-related alteration is most prominent in the frontal and cingulate cortex areas.
• Low burden is associated with more regional activation during a fine motor task.

## 1 Introduction

The mechanisms underlying brain aging are mostly unknown (Lee et al., 2022). It is suggested that single, multiple or complex interactions among structural (Bauer et al., 2021, Frey et al., 2021, Muñoz Maniega et al., 2019, Roseborough et al., 2020, Tuladhar et al., 2017, Vangberg et al., 2019), functional (Crockett et al., 2021, Guan et al., 2021, Lockhart et al., 2015) and metabolic (Bonte et al., 2017, Lee et al., 2022, Shen et al., 2012, Zhang et al., 2021) brain changes play a role. White matter hyperintensities (WMH) are one of the structural changes, measured as signal hyperintensities on T2-weighted and T2-fluid-attenuated inversion recovery (FLAIR) magnetic resonance imaging (MRI) images (Wardlaw et al., 2015). They are frequently encountered in the scans of elderly people, and may represent a major risk factor for diseases including but not limited to stroke (Aamodt et al., 2021, Schellhorn et al., 2021, Epstein et al., 2022, Longstreth et al., 2001, Schellhorn et al., 2021, Aamodt et al., 2021, Vermeer et al., 2003), dementia (Alber et al., 2019), mild cognitive impairment (Prins and Scheltens, 2015, Wardlaw et al., 2019) and Alzheimer’s disease (AD) (Hu et al., 2021) along with an additional reduction in the long-term outcomes, negative interaction with disease prognosis and recurrence (Aamodt et al., 2021, Schellhorn et al., 2021, Hu et al., 2021, Longstreth et al., 2001, Vermeer et al., 2003, Zerna et al., 2020). In this framework, understanding how WMH impact brain function in a healthy elderly population among different domains could provide insights about how these lesions add to and interact with the processes of aging as well as several neurological diseases.

Despite the fact that there are several risk factors associated with WMH including age (Garnier-Crussard et al., 2020, Grueter and Schulz, 2012, Habes et al., 2018, Moura et al., 2019), vascular conditions (Breteler et al., 1994, Grueter and Schulz, 2012, Rostrup et al., 2012, Staals et al., 2014) and sex (Lohner et al., 2022); the underlying neuropathophysiology is not fully understood. For instance, depending on the localization as periventricular WMH (pWMH) and deep WMH (dWMH), different histopathological, genetic, metabolic, cardiovascular and neuroimaging correlates may be presented (Armstrong et al., 2020, Griffanti et al., 2018, Kim et al., 2008). Furthermore, recent works have moved beyond this dichotomous subdivision and demonstrated that, with different white matter parcellation schemes, WMH spatial location may differentially contribute to cognitive functions (Jiménez-Balado et al., 2022, Wang et al., 2022), and the distributed structural damage could affect several cognitive domains (Au et al., 2006, Jiang et al., 2018b, Jiménez-Balado et al., 2022, Lampe et al., 2019, Melazzini et al., 2021, Smith et al., 2011). However, the effects of WMH’s spatial distribution are not restricted to cognition, and may also lead to alterations within the motor domain.

WMH burden has an impact on motor function which is more localized/segregated. Motor domain studies have mostly focused on the impact of WMH on lower limb motor impairment, including balance and gait, as WMH-related small vessel disease frequently has an impact on these aspects (Bolandzadeh et al., 2014, De Laat et al., 2011, Murray et al., 2010, Pinter et al., 2017, Silbert et al., 2008, Smith et al., 2015, Willey et al., 2013). In the context of WMH and upper extremity (UE) motor function, previous research has demonstrated a link for fine motor speed and muscle strength, including hand dexterity, reaction times and grip strength (Sachdev et al., 2005). A mediatory effect of WMH has also been associated with the function of the hand corresponding to the nondominant hemisphere in healthy elderly subjects (Riaz et al., 2021). Furthermore, a study with more than 700 healthy subjects with a hereditary risk factor reported a significant relationship between impaired UE performance and more WM volumes among various brain areas (Nyquist et al., 2015). Nevertheless, the research between WMH and UE is still highly limited, and to the best of our knowledge, the neural correlations of fine motor activation has been mostly unexplored. As impairments in motor function emerge both in several neurological and neurodegenerative disorders i.e stroke, Parkinson’s disease, Alzheimer’s disease and the aging process (Albers et al., 2015, Louis et al., 2005, Maetzler et al., 2015, Verghese et al., 2006), we believe a study emphasizing the role of WMH in the context of UE would help fill an important knowledge gap.

In this study, we aimed to investigate in a healthy elderly cohort whether; i) the neural activation patterns undergo changes while performing a fine motor task across different WMH distributions and burdens, ii) ultra-high field 7 T imaging would allow detecting the activation differences in specific brain areas, and iii) potential changes are also reflected by the behavioral measures. Based on this, we posit that different degrees of WMH distribution may differentially affect motor activation and performance. To investigate this, we performed a data-driven stratification of the subjects according to different distributions of periventricular WMH (pWMH) and deep WMH (dWMH) lesion volumes. Then, we compared brain activity during a finger tapping MRI task among the different groups of subjects obtained with the stratification procedure. Finally, we sought to investigate any potential brain-behavior correlation between brain activity during finger tapping task-fMRI and a clinical fine motor scale.

## 2 Methods

### 2.1 Subjects

40 elderly subjects (16 females, 24 males; mean age 69.3 years; range [56–84] years) gave written informed consent to participate in this study. The subjects were primarily included as the healthy control group of an ongoing stroke project, registered at clinicaltrials.gov (Project identifier: NCT05086055). Recruitment was done via an advertisement in the local newspaper targeting older Norwegian adults. Designated research nurses conducted a structured phone interview to make an initial assessment for the inclusion/exclusion criteria before invitation to the study. The final selection of participants was conducted physically by a designated senior medical doctor working at the Stroke Unit at St Olav’s Hospital, Trondheim. Inclusion criteria were: 1) age between 55 and 85 years; 2) no previous history of psychiatric/neurological/neurodegenerative disease; 3) no dementia; 4) no stroke; 5) no brain tumor or neurotrauma; 6) no previous musculoskeletal disorders; 7) no cognitive dysfunction; 8) no aphasia; (9) ability to give informed consent; 10) no contraindications to 7 T MR (metal in body, active implants, neurostimulator, pacemaker, cochlear implant, dental implants with metal screws, pregnancy and claustrophobia). Subjects were assessed additionally with the modified Rankin Scale (mRS) and Barthel Index (BI) and not included if they had mRS more than 2 and BI less than 95. The study conformed to the Declaration of Helsinki and was approved by the Regional Ethical Committee of Central Norway (REK number: 171264).

### 2.2 Motor clinical scale

For the clinical assessment of motor function, each subject was assessed with the 9-Hole Peg Test (9-HPT) for both left and right hand. All subjects underwent testing outside the MRI environment. The PROcare ApS 9-HPT equipment was used (dimensions of the pegs: 6.4 mm in diameter, the pegboard: 12 x 12 cm). The standardized administration instructions were followed. For each subject, the dominant hand side was measured first and the non-dominant hand side was measured second. The time to complete the test was noted.

### 2.3 MRI data acquisition

All subjects underwent MRI at 7 T (Magnetom Terra, equipped with a Nova Medical Head Coil 1TX/32RX, Siemens Healthcare GmbH, Erlangen, Germany). For mapping anatomical brain areas, we used a whole-brain coverage 3D-MP2RAGE (224 slices; FOV = 168 x 240 x 240 mm; GRAPPA acceleration factor = 3; isotropic resolution = 0.75 × 0.75 × 0.75 mm; TR = 4.3 s; TI_1_/TI_2_ = 0.84/2.37 s; FA_1_/FA_2_ = 5°/6°; TE = 1.99 ms; echo spacing = 7.2 ms; bandwidth = 250 Hz/Px; partial-fourier = 6/8; acquisition time = 9.25 min) given its high signal-to-noise ratio and gray matter/white matter contrast (Marques et al., 2010). For further identification and segmentation of WMH, we collected a 3D-FLAIR (224 slices; FOV = 168 x 202.5 x 240 mm; CAIPIRINHA acceleration factor = 3; isotropic resolution = 0.75 × 0.75 × 0.75 mm; TR = 5 s; FA = 120°; TE = 2.48 s; echo spacing = 3.92 ms; bandwidth = 651 Hz/Px; acquisition time = 6.55 min) given its sensitivity to white matter pathology detection (Wardlaw et al., 2013).

For mapping the motor areas with task-fMRI, we collected a full-brain coverage gradient-echo EPI sequence (100 slices; FOV = 198 x 198 x 150 mm; isotropic resolution = 1.5 x 1.5 x 1.5 mm; TR = 1.53 s; FA = 68°; TE = 21 ms; echo spacing = 0.77 ms; bandwidth = 1456 Hz/Px; partial-fourier = 6/8; SMS = 4; phase-encoding direction = anterior-posterior; 115 volumes). To implement susceptibility distortion correction during subsequent processing, four volumes with opposite phase-encoding direction (i.e., posterior-anterior) were acquired after each of the motor tasks.

We asked subjects to perform the motor task-fMRI finger tapping of thumbs vs. index finger with both left and right hand, in two separate runs. Order of task presentation randomized over subjects. We employed a block fMRI design with 4 blocks of rest and motor task (starting with rest) lasting 14 volumes each. A cross was omnipresent in the middle of the screen, and during motor blocks a circle started flashing around the cross at 1 Hz frequency to prompt visually cued finger tapping. See task-fMRI processing subsection for further details.

### 2.4 Structural preprocessing

First, the T1-weighted 3D-MP2RAGE and FLAIR images were defaced by using the *pydeface* toolbox (Gulban et al., 2019). Defacing was applied as a part of the data anonymization process. Then, MP2RAGE was processed using a custom modification of the SPM-based anatomical preprocessing available on https://github.com/srikash/presurfer. The pipeline has been developed and optimized for MP2RAGE preprocessing (Kashyap et al., 2021), and it used both the INV2 and UNI T1 image from the MP2RAGE sequence. Briefly, the INV2 was biascorrected and normalized as in (Choi et al., 2019) and then multiplied with the UNI to remove the salt and pepper noise pattern of the UNI image. Then, the denoised UNI image was biascorrected and segmented with SPM *NewSegment* (Kashyap et al., 2021). The same SPM-based biascorrection and segmentation was applied to FLAIR. Then, using the UNI-segmented white matter mask, the FLAIR was registered to the UNI by using boundary-based registration (Greve and Fischl, 2009). Both structural images were brain-extracted using *HD-BET*, a neural network algorithm which has been demonstrated to outperform most commonly available brain extraction algorithms (Isensee et al., 2019). However, for the UNI images the sagittal sinus was still included, which is a frequently encountered issue with 7 T MP2RAGE data. Therefore, we performed a crude manual removal of this non-brain area, as previously suggested (de Hollander et al., 2021). After manual editing, all brain masks were visually inspected to achieve optimal brain extraction results. Then, each individual UNI image was warped to the MIITRA template employing ANTS symmetric diffeomorphic registration, *anstRegistrationSyn* (Avants et al., 2008). MIITRA template is a recently defined atlas suited for use in studies on age-related brain changes and neurodegeneration (Ridwan et al., 2021). The same diffeomorphic registration step was applied to register UNI image to the MNI template. The results are needed for some of the WMH segmentation processing steps (see below).

Finally, due to the presence of WMH lesions, freesurfer *recon-all* results may have been sub-optimal or wrong (Dadar et al., 2021), particularly at the interface between white and gray matter. Therefore, we used the SLF algorithm for lesion filling (Valverde et al., 2014), which fills the WMH lesions with random intensities taken from a distribution of normal appearing white matter. This lesion-filled image together with the optimized brain mask computed at the above step were given as input to freesurfer 7.2.0 *recon-all* pipeline (Dale et al., 1999), for further anatomical processing (with options *-hires* and *-xmask*). Freesurfer outputs were used for further processing. The *recon-all* estimation of intracranial volume (eTIV) was used for normalization of individual WMH lesions volume.

### 2.5 WMH segmentation

WMH segmentation was performed with BIANCA (Griffanti et al., 2016). Both preprocessed UNI and FLAIR images were given as input to improve the classifier performance. For training BIANCA, the WMH masks of 28 subjects were manually identified on the FLAIR images by an expert neurologist, G.B. Then, BIANCA automatic segmentation was performed on the remaining subjects. Manual edits were performed when needed on the resulting WMH masks to achieve the best WMH segmentation possible. The masks were warped to MNI space using the affine matrix and warp field estimated at the ANTS normalization step (see above), using nearest neighbor interpolation (*antsApplyTranforms*). Then, each WMH mask was further subdivided into pWMH and dWMH. For identification of pWMH we follow the “contiguity-to-ventricles rule” (Griffanti et al., 2018, Melazzini et al., 2021). An MNI-space defined ventricles mask (retrieved from: https://git.fmrib.ox.ac.uk/ludovica/wmh-sub-classes) was used to identify pWMH. The mask was registered to individual native space using ANTS-estimated inverse affine matrix and warp field. WMH voxels were discretized in clusters with a minimum size of connected component of 6 voxels (fsl command *cluster* with the *connectivity* option set to 6, as in (Melazzini et al., 2021). Those clusters which overlapped, either fully or partially, with the ventricles mask, were assigned to pWMH, dWMH otherwise (Melazzini et al., 2021). This method is clinically plausible (Griffanti et al., 2018), and it avoids using a fixed distance from the ventricles to define the two lesion classes. As an example, the ventricles distance value varies in the literature according to the algorithm employed for WMH segmentation (Griffanti et al., 2016, Jiang et al., 2018a, Sundaresan et al., 2021). Finally, both classes were normalized over eTIV and log-transformed for further processing.

Furthermore, to provide a more in-depth overview of the spatial distribution of WMH, we have used the bullseye parcellation, which has also been validated with neurodegenerative disorders showing WMH lesions (Groot et al., 2018, Leijenaar et al., 2019, Sudre et al., 2017), as well as with healthy elderly populations (Jiménez-Balado et al., 2022, Salvadó et al., 2019, Sudre et al., 2018). Briefly, this white matter parcellation considers not only the distance from the ventricles, but also the anatomical (lobar) location of the lesions. The parcellation takes as input the *recon-all* results (https://github.com/gsanroma/bullseye_pipeline) and it is based on i) four equidistant concentric shells spanning the white matter from ventricles to cortex and ii) four lobar parcellation (frontal, parietal, temporal and occipital). Then, the intersection of these two parcellations is taken per hemisphere giving 32 parcels per individual (16 per each hemisphere). Furthermore, a bilateral subcortical parcellation is added (thalamus and basal ganglia), spanning the four concentric layers, resulting in a total of 36 parcels per subject.

### 2.6 Subjects’ partitioning

We stratified subjects according to the pWMH and dWMH lesion burden. We employed K-means clustering to identify the optimal number of clusters. We partitioned the subjects across different cluster values, ranging between K = 2 and K = 8, K being the number of clusters as input to the algorithm. We used 8 as the right upper extreme for the search, since it corresponds to the maximum granularity that would be obtained by the sum of the Fazekas scores, which is a 4-point clinical scale per each periventricular and deep WMH class. Then, we performed a quantification of the level of compactness and sparsity of the clusters, employing the Calinski-Harabasz metric which has been suggested for the evaluation of both the within-clusters compactness and the between-clusters separation (Liu et al., 2010). The higher the index, the lower the degree of cluster fragmentation and the higher the distinction of one cluster from the others, i.e., for higher measure of the Calinski-Harabasz index, we have better partitioning (we used the MatLab function *evalclusters*).

### 2.7 Task-fMRI preprocessing

Raw images were converted to BIDS format (*dcm2bids*). Then, the first three volumes of each task sequence were removed, resulting in a total of 112 volumes. All functional data were analyzed with *fmriprep* (Esteban et al., 2019), version 22.0.2, see supplementary materials for the details of the analysis with the fmriprep-generated boilerplate.

All subsequent preprocessing steps were performed in MIITRA elderly template space. First, brain extraction was performed using the brain mask estimated with *fmriprep*. Then, all the next steps were performed with FSL (Jenkinson et al., 2012) (FMRIB's Software Library, https://fsl.fmrib.ox.ac.uk/fsl/fslwiki) as implemented with FEAT (Woolrich et al., 2009), including spatial smoothing (Gaussian kernel, FWHM = 3 mm), grand-mean intensity normalization of all volumes and *fmriprep*-estimated confounds by a single multiplicative factor, as suggested in (Waller et al., 2022), and, finally high-pass temporal filtering (Gaussian-weighted least-squares straight line fitting, sigma = 45 s).

### 2.8 Task-fMRI analysis

*First level analysis.* To detect task-related motor activity, one explanatory variable (EV) was defined to model the On-Off periods of the task per each run (left or right finger tapping) and convolved with a double-gamma hemodynamic response function. The 6 motion parameters (three head rotations and three head translations), framewise displacement (FD), mean cerebrospinal fluid and mean white matter signals were added to the general linear model (GLM) as covariates of no interest. We also performed motion scrubbing, by removing all volumes which exceeded a FD value of 0.5 mm from the analysis, as calculated in the *fmriprep*-generated confounds file.

*Second level analysis - group activity.* To model group mean activation, one sample *t*-test was used per task (left and right finger tapping) and group of subjects. To model between-group activation, we used two-sample unpaired *t*-test. Results were converted to *z*-values and then thresholded at Z ≥ 3.1 for activity cluster formation, followed by a significance threshold of p = 0.001 (cluster corrected using Gaussian Random Field Theory). For all the analyses, we added age, sex, education, handedness, and mean FD as covariates. Finally, the Z-threshold maps were further resampled to a 0.5 mm resolution version of the MIITRA template (Niaz et al., 2022).

### 2.9 Brain-behavior correlation: Brain activity with motor performance

The correlation between brain activation during finger tapping and the clinical measure of 9-HPT were modeled with age, sex, education, handedness, and mean FD values as covariates. Z-maps were thresholded at Z ≥ 3.1 for cluster formation, followed by with a significance threshold of p = 0.001 (cluster corrected using Gaussian Random Field Theory).

### 2.10 Brain-behavior correlation: WMH lesion load and motor performance

In addition to the analysis between the clinical measures of 9-HPT and the brain activity during finger tapping fMRI, we sought to investigate the potential relationship between the total WMH, pWMH, dWMH lesion load (normalized against ICV and log-transformed to improve gaussianity); and the 9-HPT. We used multiple linear regression (MLR) employing the function *nc_FitAndEvaluateModels* (https://github.com/jyeatman/lifespan). We fitted one model per each burden type. We first regressed out age, sex, handedness, and education from the dependent variable. Then we used the residuals from the previous step to regress against each modality of WMH burden. Then, we estimated the accuracy of the linear regression (residuals against WMH burden) using leave-one-out cross validation (LOOCV) and we calculated the coefficient of determination (R^2^) from this procedure (Faskowitz et al., 2018). To obtain an empirically estimated p-value, we randomly permuted WMH burden across 5000 ordinary least-squares fit iterations. We applied Bonferroni correction, which resulted in an alpha value of 0.0014 (0.0083/6, three modalities of WMH burdens and two hand sides).

## 3 Results

The demographic, clinical and MRI characteristics of the sample and the clusters are reported in Table 1 and Table 2. Of the 40 subjects included, two resulted in severe head-motions during task performance, and one did not complete the MRI exam and only the structural scans were acquired, resulting in a sample size of 37 subjects for task-fMRI analysis.

**Table 1:** Demographic and clinical information of the sample and the groups.

	**Group 1 (N = 6)**	**Group 2 (N = 18)**	**Group 3 (N = 16)**	**Full sample (N = 40)**
**Demographics**				
Age (mean years, SD)	68.7 (7.5)	65.8 (6.2)	73.6 (6.4)	69.3 (7.3)
Sex (female/male)	4F, 2 M	5F, 13 M	7F, 9 M	16F, 24 M
Education (mean years, SD)	15.8 (4.5)	17.3 (2.9)	15.7 (2.5)	16.5 (3.1)
Handedness (right/left)	6R	15R, 3L	15R, 1L	36R, 4L
**Risk Factors**				
Hypertension	1	3	5	9
Atrial fibrillation	0	2	3	5
Diabetes mellitus	0	1	0	1
Previous smoking*	2	7	2	11
**Medical Assessments**				
modified Rankin scale (mean score 0 – 5)	0.1	0.1	0.1	0.1
Barthel index (mean score, 0 – 100)	99.1	99.7	99.6	99.6
**Cognitive Assessment**				
Montreal cognitive assessment (mean score, SD 0–30)	27 (0.8)	26.4 (1.9)	24.4 (3.6)	25.7 (2.8)
**Motor Scale**				
9-hole peg test right (mean seconds, SD)	14.9 (3.1)	14.8 (2)	17.3 (3.3)	15.8 (2.9)
9-hole peg test left (mean seconds, SD)	15.6 (2.5)	15.5 (2)	18.1 (3.2)	16.6 (2.8)
9-hole peg test difference (mean seconds, SD)	0.9 (0.6)	1.8 (2)	2 (2)	1.8 (1.9)

**Table 2:** MRI characteristics of the sample and the groups.

**[ml]**	**Median**	**IQR**	**Min**	**Max**	
**Group 1**	**pWMH**	0.78	1.03	0.32	1.93
	**dWMH**	0.02	0.02	0.00	0.03
**Group 2**	**pWMH**	0.99	1.52	0.25	2.86
	**dWMH**	0.17	0.13	0.05	0.35
**Group 3**	**pWMH**	4.96	11.64	1.84	19.05
	**dWMH**	1.11	2.09	0.50	4.84
**Full sample**	**pWMH**	1.93	2.81	0.25	19.05
	**dWMH**	0.25	0.70	0.00	4.84
	**ICV**	1025.4	166.1	771.7	1704.1

*IQR: interquartile range, dWMH: deep white matter hyperintensities, pWMH: peripheral white matter hyperintensities, ICV: intracranial volume.

### 3.1 WMH lesion load at 7 T

**Fig. 1:**
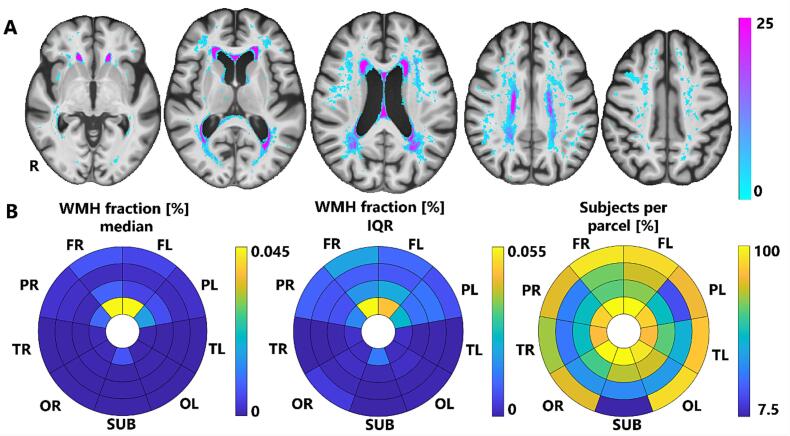
**Spatial distribution of WMH. A.** Spatial distribution of WMH at 7 T MRI. Fractions of subjects over the entire sample (%, N = 40) are represented. The resulting map is overlaid onto the MIITRA elderly template (axial slices are Z = 2, 13, 24, 34, 43, from left to right). **B**. WMH fraction distribution (normalized over ICV) indicated by the median (leftmost) and IQR (middle) values, respectively. The rightmost bullseye plot depicts how many subjects (as a fraction of the whole sample, %) showed a WMH lesion in each of the bullseyes’ parcels.

WMH macro-scale spatial distribution is depicted in Fig. 1. WMH were predominant in the frontal and parietal lobe bilaterally, where the number of lesions decreased as a function of the distance from the ventricles (highest amount) to cortex (lowest amount). The bullseyes plots (see Fig. 1B) indicate that in frontal and parietal areas and in the layers closer to the ventricles the number of lesions is the highest, but also the most variable (as shown by the inter quartile range (IQR) values). Lesion burden is also common in subcortical areas, but again only in the parcel closest to the ventricular system. All the remaining subcortical areas, temporal and occipital lobes (with a very mild effect of the ventricles’ distance), had a lower extent of WMH lesion load. An association between the WHM lesion load and task-related activation was found for the left hemisphere.

### 3.2 Subjects’ clustering

**Fig. 2:**
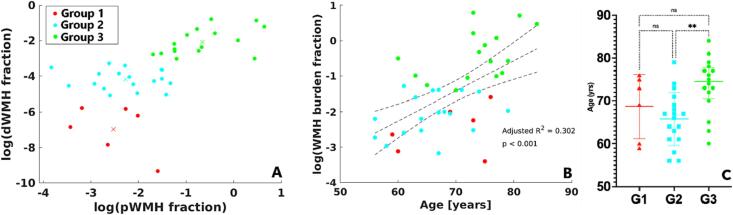
**A.** Subjects’ clustering according to pWMH and dWMH burden. Scatter plot depicting the results of the unsupervised K-means algorithm for the best number K = 3 partitions (highest Calinski-Harabasz index). Group 1 (G1, red dots, small dWMH and low-to-mild pWMH), Group 2 (G2, cyan dots, mild dWMH and low-to-mild pWMH) and Group 3 (G3, green dots, both heavy pWMH and dWMH). Crosses represent the estimated centroid for each of the estimated partitions. **B.** Linear relationship between age and WMH burden. Dotted black lines represent the linear fit to the data and 95 % confidence intervals for the fit. Each subject’s group membership is color-coded using the same colors as in panel A. **C.** Age distribution for clusters G1, red triangles; G2, cyan squares; and G3, green circles. Significant difference between G2 and G3 (p < 0.01). (For interpretation of the references to color in this figure legend, the reader is referred to the web version of this article.)

We obtained the best subjects’ partitioning at K = 3 clusters, corresponding to the highest Calinski-Harabasz index. Therefore, we subdivided our population into three groups, corresponding to different levels of pWMH and dWMH burdens. We will refer to these three partitions as Group 1 (G1), Group 2 (G2) and Group 3 (G3), see Fig. 2A. G1 and G2 presented the same amount of pWMH (low to mild), but different dWMH (G1: low dWMH burden; and G2: mild dWMH burden), while G3 showed a high lesion load in terms of both dWMH and pWMH. Hence, the dWMH burden acted as the discriminative factor between G1 and G2, while pWMH remains on the same burden level, from low to mild. On the other hand, subjects showing heavy dWMH and pWMH burdens were merged into the same cluster by the K-means algorithm. We found a significant linear association (Fig. 2B) between age and the total WMH burden. A significant difference in age was further found between G2 and G3 (p < 0.01) (Fig. 2C). Due to the imbalanced number of males/females in the groups, all the analyses conducted were adjusted for age and sex.

### 3.3 Effect of WMH burden on motor performance: Between-group comparisons

**Fig. 3:**
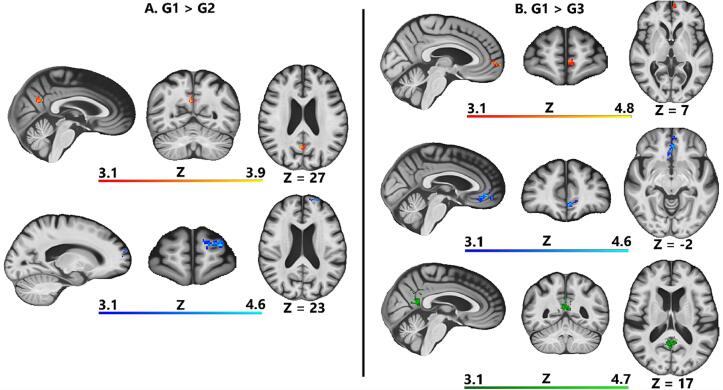
**Cluster of higher neural activity while contrasting G1 vs G2 and G3. A.** G1 > G2. A total of two clusters were statistically significant. Top row: midline cluster (red-yellow) of increased activation spanning left and right PRE, and left RSC. Bottom row: lateralized left hemisphere cluster (blue-light blue) showing activity in MFC, SFC. **B.** G1 > G3. A total of three separate networks of activity resulted after group level comparison. Top row: MFC and ACC (red-yellow). Middle row: midline cluster MFC, ACC, caudate nucleus (blue-light blue). Bottom row: another midline cluster (green) showing activity spanning PCC, PRE, RSC bilaterally. All the results are cluster-corrected for multiple comparisons (Z ≥ 3.1, p < 0.001) and are shown overlaid on the MIITRA elderly template. PRE: precuneus, RSC: retrosplenial cortex, MFC: middle frontal cortex, SPC: superior frontal cortex, ACC: anterior cingulate cortex, PCC: posterior cingulate cortex. (For interpretation of the references to color in this figure legend, the reader is referred to the web version of this article.)

To explore differences in brain activity among groups i.e., to investigate if different WMH burdens affected the brain activity during motor tasks differently, we performed the following comparisons: (i) G1 > G2 and G2 > G1; (ii) G1 > G3 and G3 > G1; (iii) G2 > G3 and G3 > G2. Each group comparison has been performed for both left and right finger tapping separately. All the following results referred to right finger tapping. The G1 > G2 contrast revealed two clusters of significant BOLD signal change spanning different regions: the left/right precuneus (PRE) and left retrosplenial cortex (RSC) (see Fig. 3A, top row, red-yellow cluster). A second left lateralized cluster covered the middle and superior frontal cortex (MFC and SFC) (see Fig. 3A, bottom row, blue-light blue cluster). The G1 > G3 contrast resulted in one frontal cluster of activation, spanning bilaterally the medial portion of SFC and the anterior cingulate cortex (ACC), as well as the left orbitofrontal cortex (OFC) (see Fig. 3B, top row, red-yellow cluster). A second cluster of greater activation of G1 with respect to G3 was again found in the OFC, ACC; but extending more ventrally with respect to the previous cluster and including small portion of the right caudate nucleus (see Fig. 3B, middle row, blue light-blue cluster). Lastly, a third bilateral cluster was localized in left and right posterior cingulate cortex (PCC), PRE and RSC (see Fig. 3B, bottom row, green cluster). Instead, when contrasting whether G2 and G3 activated less than G1 (i.e., G1 < G2 and G1 < G3), no significant activation survived correction for multiple comparisons. The remaining group contrasts (G2 > G3 and G3 > G2) resulted in no significant activation maps. As for left finger tapping no activations maps differences were found.

### 3.4 Relationship between 9-HPT and brain activity

The MLR models between 9-HPT and the brain activity during finger tapping, adjusted for age, sex, years of education and handedness, did not show significant association in any of the groups of subjects, neither for right nor left thumb vs index tapping.

### 3.5 Relationship between lesion load and 9-HPT

The MLR models between three types of WMH burden (total, peri and deep WMH volume) and 9-HPT did not result in any significant parameter estimates (Bonferroni correction, α = 0.0014), after adjusting for age, sex, years of education and handedness. The same analysis was performed for each of the subject’s groups separately, stratified according to the K-means algorithm, resulting in no significant associations after correction for multiple comparisons.

## 4 Discussion

How the extent of WMH lesion can affect brain activity in a population of healthy elderly subjects performing a motor task is currently not fully understood. To try to answer this question, we stratified our subjects according to their WMH spatial location and burden and compared the brain activity during finger-tapping fMRI task. We saw that the low burden group demonstrated more activation in areas belonging to a frontal network (MFC, SFC), ACC, PRE during the right finger tapping task.

### 4.1 WMH lesion load’s impact on motor activity

WMH lesion load primarily affects the myelin structure and subsequently the axons’ integrity (Wakita et al., 2002, Wardlaw et al., 2015). This is likely to result in rerouting via other cortical areas and alterations in neural connections. G2 and G3 showed reduced activity in prefrontal and cingulate regions when compared to G1. This could occur due to the high extent of WMH-induced disconnections, preventing healthy recruitment of those areas, assuming that G1 is the group with the most intact underlying mechanisms. Additionally, more activation in specified brain areas has also been referred to as “repetition enhancement” and complies with the nature of the finger tapping task (Eryurek et al., 2022). Since the subjects in the low burden group have more intact connections, healthier recruitment mechanisms are likely to result in more activation in multiple brain areas. The observed association between degree of WMH with damage to the axons, that comes in addition to the myelin damage documented with a lesser load of WMH, may indicate that the load of WMH burden slows down communication and causes a rerouting of subsequent signals between brain centers, as also suggested in the literature (Wakita et al., 2002, Wardlaw et al., 2015).

The increased activity in some frontal areas, such as MFC, SFC and OBC, in G1 subjects, compared to subjects in G2 and G3 correspond to the areas that have been associated with age-related metabolic decline (Lee et al., 2022). Moreover, WMH structural damage in the frontal lobe has a detrimental effect on executive functions (Jiménez-Balado et al., 2022, Smith et al., 2011). Our findings provide further support of the importance of the frontal lobes in WMH pathophysiology, by providing new evidence of dysfunctional motor-related neural correlates which are likely attributable to WMH.

Interestingly, in our study we found increased activation in PCC and MFC for the low burden group. These areas overlap with some of the default mode network (DMN) core nodes (Raichle et al., 2001, Shulman et al., 1997). DMN and motor networks have been priorly associated with motor readiness and movement preparation (Bazán et al., 2015). Previous studies (Berlot et al., 2020, Gobel et al., 2011) on motor sequence learning showed more activation in DMN regions of MFC, PCC and adjacent ventral precuneus areas in the initial learning phase. This line of evidence supports our findings and proposes the suppression of DMN deactivation as a possible impaired mechanism within this work. The finger tapping motor task used in this study consists of sequential movement patterns and requires both motor readiness and movement preparation. Therefore, when WMH burden is low, the reduced DMN suppression mechanism, i.e., more activation in DMN, is likely to be intact and it is indeed shown only in the G1 group. Conversely, in G2 and G3, the activity suppression of DMN nodes is presumably impaired, and the activity in DMN is decreased (Mason et al., 2007, Weissman et al., 2006).

Furthermore, the deactivation of DMN has previously been suggested in tasks that require attention (Raichle et al., 2001). Studies also reported activation of DMN during sustained-attention tasks, specifically when the task is considered mundane and prone to mind-wandering (Danckert and Merrifield, 2018). The finger tapping task in a block design is repetitive and fails to captivate long-term enthusiasm, does not have a high cognitive demand, yet entails sustained attention. Moreover, this decreased deactivation among DMN structures has also been linked to enhanced cognitive capacity and elevated neural effectiveness for healthy older subjects. Additionally, amnestic mild cognitive impairment and Alzheimer’s disease patients with higher cognitive capacity have been shown to have an opposite activation pattern, more deactivation in particularly PCC and anterior cingulate regions, in comparison with the patients lower cognitive capacities (Mevel et al., 2011). These studies overall could help explain the increased activation in some of the DMN nodes for the low burden group, whereas the most affected groups having less neural effectiveness could rely on altered networks to perform the task due to WMH lesions. Nevertheless, DMN is a complex network, and the extent of its functions is yet to be fully understood in the healthy and the diseased brain, requiring cautious interpretation of the results.

Finally, we did not find any differences in areas which are typically related to motor control such as primary motor cortex, supplementary motor cortex, cerebellum, and basal ganglia; except for a small portion of the caudate nucleus. Previous PET studies have shown that those areas remain both metabolically and structurally intact (Petit-Taboue et al., 1998, Zimmerman et al., 2021), despite increasing age. Presumably, WMH-mediated white matter damage does not influence these areas in the healthy elderly, corroborating previous findings with motor task-fMRI data.

### 4.2 Motor clinical scale

The MLR models did not show any significant results, neither for task fMRI nor for WMH volumetric data. This is not surprising, since our population consists of healthy elderly subjects, who can perform the task and follow the instructions for the finger tapping paradigm well. A previous study investigated the relationship between WMH and finger tapping performance as measured on how many taps a subject could execute in one minute, outside the MRI environment. As in our study, the authors did not report any significant correlations (Silbert et al., 2008). Conversely, another study examined the contribution of physical activity to the development of WMH burden (Fleischman et al., 2015). The authors reported a significant association between WMH lesion load and motor function, measured with a composite score, which pooled different measurements together. The reason behind this mismatch can be identified with different sample sizes in our study and previous studies. Moreover, the scales employed to assess motor dexterity are different from our study, which limits the comparison of results.

Last but not least, we would like to emphasize the complexity of the statistical adjustment process and address potential inherent limitations that come with adjusting for age and sex. As stated in previous literature, adding age and sex as covariates does not serve as a comprehensive solution for between-group comparisons, and cannot fully account for the potential confounding factors such as the residuals and other unknown interactions that may lead to overestimating the precision (Miller and Chapman, 2001). Particularly, the aging process and WMH are not mutually exclusive, but they interact with each other on multiple levels. The methods and data analysis steps in this study are chosen to primarily reflect the differences in WMH distribution and burden, nevertheless, the interpretation of the findings should be done while taking potential limitations into consideration.

## 5 Clinical implications and future directions

In this study, we chose to focus on motor function, since decreased motor ability affects both the healthy elderly (Louis et al., 2005, Verghese et al., 2006) as well as patients affected by a stroke, vascular dementia and neurodegenerative diseases including Parkinson’s (Maetzler et al., 2015) and Alzheimer’s disease (Albers et al., 2015). Our findings suggest that at a preclinical stage, different WMH burdens may lead to changes in the recruitment of neural connections during an upper-extremity (UE) fine motor task. These WM-related differences in UE are likely to be present in patients as well and could easily be overlooked in the evaluation of more acute and of pronounced pathologic changes in the cognitive and lower extremity motor domains. As WMH is a major risk factor with many diseases that also include UE motor symptoms such as stroke, we suggest that investigating the association with UE function further in patient and/or pathological aging studies could provide additional insights in understanding WMH pathology.

Secondly, the highlighted brain areas could be examined in future studies of patients with different degrees of motor symptoms. The activity/connectivity, structural features and network properties of these brain areas could be followed longitudinally to further define their relevance; as they could be the initial regions to present alterations at the preclinical stages and could be potential targets for motor function enhancement. The gained information could be particularly useful for the diseases with motor symptoms. Several studies reported promising intervention effects for stroke and Alzheimer’s disease through non-invasive neurostimulation protocols (Ahmed et al., 2023, Banduni et al., 2023, Kong et al., 2023, Williams et al., 2009). These brain regions have in the future the potential to be targeted, after their relevance to upper extremity motor symptoms in patients have been assessed further.

Neuroimaging in this regard becomes a powerful aid, as WMH do not follow a stable course over time and are associated with several underlying conditions including secondary impairments in the cortex, harm to long tracts and decreasing overall brain health (Wardlaw et al., 2015). There is an extensive body of evidence that indicates the significance of WMH and the important role these lesions play in both aging, cognition, and neurodegenerative processes. WMH may be considered as a biomarker and serve to identify otherwise healthy, asymptomatic individuals who may be at increased risk of developing neurological diseases. Follow up of these individuals could enable timely prevention strategies such as initiation of prophylactic treatment and/or behavioral changes that could reduce the overall burden of disease for these subjects.

## 6 Conclusions

In summary, we investigated the impact of different WMH spatial distributions on the neural correlates of fine motor control and found differences in neural activation among groups with different WMH burdens particularly in the frontal regions. It is important to identify these alterations in the healthy elderly, as they could in the future potentially serve as biomarkers for treatment and prevention strategies for several neurodegenerative diseases.

## CRediT authorship contribution statement

**Riccardo Iandolo:** Conceptualization, Data curation, Formal analysis, Methodology, Writing – original draft, Visualization. **Esin Avci:** Conceptualization, Data curation, Methodology, Writing – review & editing. **Giulia Bommarito:** Formal analysis. **Ioanna Sandvig:** Conceptualization, Resources, Supervision, Writing – review & editing. **Gitta Rohweder:** Conceptualization, Data curation, Supervision, Writing – review & editing. **Axel Sandvig:** Conceptualization, Funding acquisition, Methodology, Project administration, Resources, Supervision, Validation, Writing – review & editing.

## Declaration of competing interest

The authors declare that they have no known competing financial interests or personal relationships that could have appeared to influence the work reported in this paper.

## Data Availability

Raw and processed MRI data that have been used are confidential. Other data will be made available upon reasonable request.
