# Supplementary material for: Characterizing upper extremity fine motor function in the presence of white matter hyperintensities: A 7 T MRI cross-sectional study in older adults
Source: Neuroimage Clin. 2024 Jan 24;41:103569. doi: 10.1016/j.nicl.2024.103569 (PMC10839532; doi:10.1016/j.nicl.2024.103569)
Supplement: Supplementary Data 1 [file mmc1.docx]

**Supplementary Materials**

***Fmriprep boilerplate***

Results included in this manuscript come from preprocessing performed using *fMRIPrep* 22.0.2 (@fmriprep1; @fmriprep2; RRID:SCR_016216), which is based on *Nipype* 1.8.5 (@nipype1; @nipype2; RRID:SCR_002502).

***Preprocessing of B0 inhomogeneity mappings***

A total of 5 fieldmaps were found available within the input BIDS structure for this particular subject. A *B0*-nonuniformity map (or *fieldmap*) was estimated based on two (or more) echo-planar imaging (EPI) references with `topup` (@topup; FSL 6.0.5.1:57b01774).

***Anatomical data preprocessing***

A total of 1 T1-weighted (T1w) images were found within the input BIDS dataset. The T1-weighted (T1w) image was corrected for intensity non-uniformity (INU) with `N4BiasFieldCorrection` [@n4], distributed with ANTs 2.3.3 [@ants, RRID:SCR_004757], and used as T1w-reference throughout the workflow. Brain tissue segmentation of cerebrospinal fluid (CSF), white-matter (WM) and gray-matter (GM) was performed on the brain-extracted T1w using `fast` [FSL 6.0.5.1:57b01774, RRID:SCR_002823, @fsl_fast]. Volume-based spatial normalization to three standard spaces (MIITRA, MNI152NLin2009cAsym, MNI152NLin6Asym) was performed through nonlinear registration with `antsRegistration` (ANTs 2.3.3), using brain-extracted versions of both T1w reference and the T1w template. The following templates were selected for spatial normalization: *MIITRA template for elderly and neurodegeneration study, see <https://onlinelibrary.wiley.com/doi/full/10.1002/hbm.25327> and https://www.sciencedirect.com/science/article/pii/S105381192101140X* [@miitra, RRID:SCR_002823; TemplateFlow ID: MIITRA], *ICBM 152 Nonlinear Asymmetrical template version 2009c* [@mni152nlin2009casym, RRID:SCR_008796; TemplateFlow ID: MNI152NLin2009cAsym], *FSL's MNI ICBM 152 non-linear 6th Generation Asymmetric Average Brain Stereotaxic Registration Model* [@mni152nlin6asym, RRID:SCR_002823; TemplateFlow ID: MNI152NLin6Asym].

***Functional data preprocessing***

For each of the 2 BOLD runs found per subject (across all tasks and sessions), the following preprocessing was performed. First, a reference volume and its skull-stripped version were generated using a custom methodology of *fMRIPrep*. Head-motion parameters with respect to the BOLD reference (transformation matrices, and six corresponding rotation and translation parameters) are estimated before any spatiotemporal filtering using `mcflirt` [FSL 6.0.5.1:57b01774, @mcflirt]. The estimated *fieldmap* was then aligned with rigid-registration to the target EPI (echo-planar imaging) reference run. The field coefficients were mapped on to the reference EPI using the transform. BOLD runs were slice-time corrected to 0.744s (0.5 of slice acquisition range

0s-1.49s) using `3dTshift` from AFNI [@afni, RRID:SCR_005927]. The BOLD reference was then co-registered to the T1w reference using `mri_coreg` (FreeSurfer) followed by `flirt` [FSL 6.0.5.1:57b01774, @flirt] with the boundary-based registration [@bbr] cost-function. Co-registration was configured with twelve degrees of freedomto account for distortions remaining in the BOLD reference. Several confounding time-series were calculated based on the *preprocessed BOLD*: framewise displacement (FD), DVARS and three region-wise global signals. FD was computed using two formulations following Power (absolute sum of relative motions, @power_fd_dvars) and Jenkinson (relative root mean square displacement between affines, @mcflirt). FD and DVARS are calculated for each functional run, both using their implementations in *Nipype* [following the definitions by @power_fd_dvars]. The three global signals are extracted within the CSF, the WM, and the whole-brain masks. Additionally, a set of physiological regressors were extracted to allow for component-based noise correction [*CompCor*, @compcor]. Principal components are estimated after high-pass filtering the *preprocessed BOLD* time-series (using a discrete cosine filter with 128s cut-off) for the two *CompCor* variants: temporal (tCompCor) and anatomical (aCompCor). tCompCor components are then calculated from the top 2% variable voxels within the brain mask. For aCompCor, three probabilistic masks (CSF, WM and combined CSF+WM) are generated in anatomical space. The implementation differs such that instead of eroding the masks by 2 pixels on BOLD space, a mask of pixels that likely contain a volume fraction of GM is subtracted from the aCompCor masks. This mask is obtained by thresholding the corresponding partial volume map at 0.05, and it ensures components are not extracted from voxels containing a minimal fraction of GM. Finally, these masks are resampled into BOLD space and binarized by thresholding at 0.99 (as in the original implementation). Components are also calculated separately within the WM and CSF masks. For each CompCor decomposition, the *k* components with the largest singular values are retained, such that the retained components' time series are sufficient to explain 50 percent of variance across the nuisance mask (CSF, WM, combined, or temporal). The remaining components are dropped from consideration. The head-motion estimates calculated in the correction step were also placed within the corresponding confounds file. The confound time series derived from head motion estimates and global signals were expanded with the inclusion of temporal derivatives and quadratic terms for each [@confounds_satterthwaite_2013]. Frames that exceeded a threshold of 0.5 mm FD or 1.5 standardized DVARS were annotated as motion outliers. Additional nuisance timeseries are calculated by means of principal components analysis of the signal found within a thin band (*crown*) of voxels around the edge of the brain, as proposed by [@patriat_improved_2017]. The BOLD time-series were resampled into standard space, generating a *preprocessed BOLD run in MIITRA space*. First, a reference volume and its skull-stripped version were generated using a custom methodology of *fMRIPrep*. Automatic removal of motion artifacts using independent component analysis [ICA-AROMA, @aroma] was performed on the *preprocessed BOLD on MNI space* time-series after removal of non-steady state volumes and spatial smoothing with an isotropic, Gaussian kernel of 6mm FWHM (full-width half-maximum). Corresponding "non-aggresively" denoised runs were produced after such smoothing. Additionally, the "aggressive" noise-regressors were collected and placed in the corresponding confounds file. All resamplings can be performed with *a single interpolation step* by composing all the pertinent transformations (i.e. head-motion transform matrices, susceptibility distortion correction when available, and co-registrations to anatomical and output spaces). Gridded (volumetric) resamplings were performed using `antsApplyTransforms` (ANTs), configured with Lanczos interpolation to minimize the smoothing effects of other kernels [@lanczos]. Non-gridded (surface) resamplings were performed using `mri_vol2surf` (FreeSurfer).

Many internal operations of *fMRIPrep* use *Nilearn* 0.9.1 [@nilearn, RRID:SCR_001362], mostly within the functional processing workflow. For more details of the pipeline, see [the section corresponding to workflows in *fMRIPrep*'s documentation](https://fmriprep.readthedocs.io/en/latest/workflows.html "FMRIPrep's documentation").

**### Copyright Waiver**

The above boilerplate text was automatically generated by fMRIPrep with the express intention that users should copy and paste this text into their manuscripts *unchanged*. It is released under the [CC0](https://creativecommons.org/publicdomain/zero/1.0/) license.

**Group activity during finger tapping task-fMRI**

**
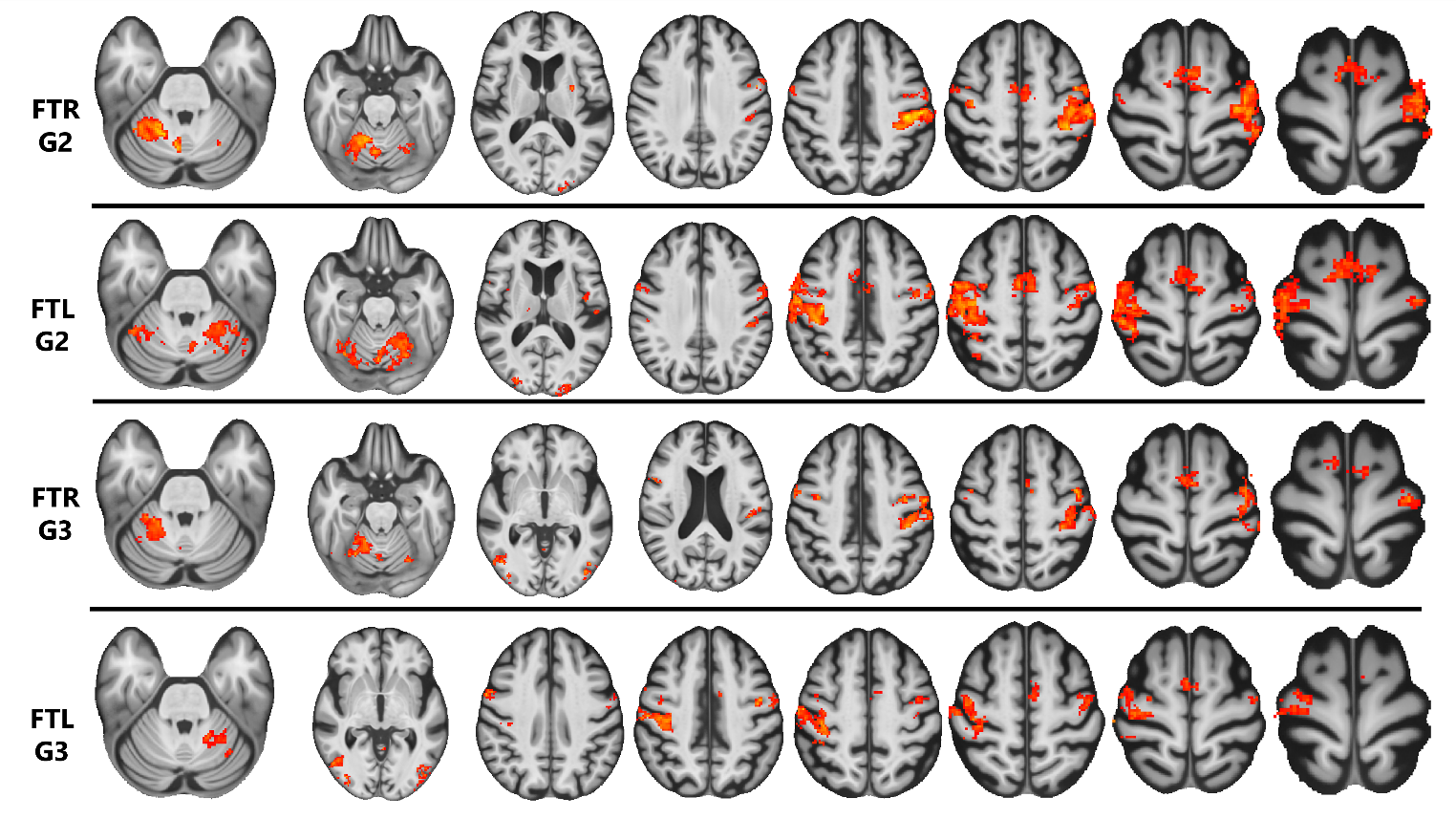
**

**Figure Supplementary 1.** Group activity for finger tapping right (FTR) and finger tapping left (FTL) in group 2 and group 3. Groups are showing mild dWMH and low-to-mild pWMH (G2) and high pWMH/dWMH burden (G3). All activation maps are cluster-corrected for multiple comparison (Z ≥ 3.1, p < 0.001) and are shown overlaid on the MIITRA elderly template. Group 1 did not show any cluster of statistically significant activity at this Z-threshold (Z ≥ 3.1) and p-value (p < 0.001)
